# Palmitoylation of the Immunity Related GTPase, Irgm1: Impact on Membrane Localization and Ability to Promote Mitochondrial Fission

**DOI:** 10.1371/journal.pone.0095021

**Published:** 2014-04-21

**Authors:** Stanley C. Henry, Elyse A. Schmidt, Michael B. Fessler, Gregory A. Taylor

**Affiliations:** 1 Geriatric Research, Education, and Clinical Center, VA Medical Center, Durham, North Carolina, United States of America; 2 Departments of Medicine; Molecular Genetics and Microbiology; and Immunology; Division of Geriatrics, and Center for the Study of Aging and Human Development, Duke University Medical Center, Durham, North Carolina, United States of America; 3 Laboratory of Respiratory Biology, NIEHS, National Institutes of Health, Research Triangle Park, North Carolina, United States of America; Ecole Polytechnique Federale de Lausanne, Switzerland

## Abstract

The Immunity-Related GTPases (IRG) are a family of large GTPases that mediate innate immune responses. Irgm1 is particularly critical for immunity to bacteria and protozoa, and for inflammatory homeostasis in the intestine. Although precise functions for Irgm1 have not been identified, prior studies have suggested roles in autophagy/mitophagy, phagosome remodeling, cell motility, and regulating the activity of other IRG proteins. These functions ostensibly hinge on the ability of Irgm1 to localize to intracellular membranes, such as those of the Golgi apparatus and mitochondria. Previously, it has been shown that an amphipathic helix, the αK helix, in the C-terminal portion of the protein partially mediates membrane binding. However, in absence of αK, there is still substantial binding of Irgm1 to cellular membranes, suggesting the presence of other membrane binding motifs. In the current work, an additional membrane localization motif was found in the form of palmitoylation at a cluster of cysteines near the αK. An Irgm1 mutant possessing alanine to cysteine substitutions at these amino acids demonstrated little residual palmitoylation, yet it displayed only a small decrease in localization to the Golgi and mitochondria. In contrast, a mutant containing the palmitoylation mutations in combination with mutations disrupting the amphipathic character of the αK displayed a complete loss of apparent localization to the Golgi and mitochondria, as well as an overall loss of association with cellular membranes in general. Additionally, Irgm1 was found to promote mitochondrial fission, and this function was undermined in Irgm1 mutants lacking the palmitoylation domain, and to a greater extent in those lacking the αK, or the αK and palmitoylation domains combined. Our data suggest that palmitoylation together with the αK helix firmly anchor Irgm1 in the Golgi and mitochondria, thus facilitating function of the protein.

## Introduction

The Immunity Related GTPases (IRG) are a family of vertebrate proteins that, like the related Guanylate Binding proteins (GBPs) and the Mx proteins, mediate innate immunity to a variety of pathogens [1]–[3]. Mice that lack expression of IRGs display decreased host resistance, though the impact on resistance and the spectra of pathogens that are involved varies depending on the IRG protein. IRGs are divided into subfamilies, with the IRGM subfamily appearing to play the most important role in host resistance [4]. Absence of IRGM proteins in mice - and Irgm1 in particular - leads to profound susceptibility to many bacteria and protozoa (e.g. *Salmonella typhimurium, Listeria monocytogenes, Mycobacterium tuberculosis, and Toxoplasma gondii*), compared to the relatively weak or normal susceptibility seen when IRGA, IRGB, and IRGD subfamily proteins are lacking [5]–[8]. In humans, genome wide association (GWAS) studies have established that the human *IRGM* gene is a susceptibility allele for both Crohn’s Disease [9], [10] and *M. tuberculosis* infection [11], [12].

IRGs mediate cell autonomous control of pathogen growth in both hematopoietic and non-hematopoietic cells. The underlying mechanism(s) are not completely clear, but involve IRG-mediated assembly on, and likely modulation of, various intracellular membranes. One well-studied example is the restriction of the growth of intracellular *Toxoplasma gondii* [13]–[15]. In uninfected cells, IRG proteins from the IRGA, IRGB, and IRGD subfamilies are distributed among distinct intracellular lipid membrane compartments [16], [17]. However, once a host cell is infected with *T. gondii*, the IRGs rapidly relocalize to the parasitophorous vacuole, where they drive vesiculation of the vacuole, releasing the parasite into the cytosol, and enabling its eradication [15]. Because IRGs are known to dimerize and multimerize in a GTP-dependent fashion [18], one possibility is that they may function like dynamins in this context in being able to contract and exert a mechanical force on the parasitophorous vacuole that prompts its vesiculation [19].

While Irgm1 is a critical requirement for the IRG *T. gondii* clearance function, it is not among the IRGs that relocalize to the *T. gondii* vacuole and participate directly in vesiculation [16], [20]. An explanation for this seeming paradox lies in the role of Irgm1 (and other IRGMs) as global regulators of other subfamilies of IRGs (‘effector’ IRGs), exerting control by governing the positioning of the effector IRGs in the cell and thus their activities [21]–[23]. Irgm1 is thought to do so by localizing to several intracellular membrane compartments where it can block IRG recruitment and therefore functioning on those membranes. Beyond this regulatory function Irgm1 possesses other activities centered on membranous compartments that impact host resistance to pathogens; these include (a) modulating autophagy and mitophagy [24], [25], (b) driving cell motility [26], (c) regulating the recruitment of non-IRG factors to pathogen-containing vacuoles that presumably control vacuole processing (e.g. snapin) [27], and (d) regulating mitochondrial fission (current manuscript). While much is to be clarified regarding the activities of Irgm1, it is seems likely that the ability of it and the other IRGs to exert their functions hinges on their ability to bind intracellular membranes.

The mechanisms through which IRGs bind membranes are incompletely defined. Irga6 is known to bind membranes via myristoylation [17]. In contrast, Irgm1 lacks myristoylation, but possesses an amphipathic helix in the C-terminus of the protein - the ‘αK’ helix – that in part directs binding [17], [20], [27]. The protein contains at least one additional membrane binding domain, however, as a mutant of Irgm1 in which the αK has been rendered nonfunctional retains some membrane binding [17]. The studies presented here seek to identify an additional mechanism through which Irgm1 binds intracellular membranes.

## Materials and Methods

### Mice and Cell Culture

Knockout C57Bl/6 mice deficient for Irgm1 (Irgm1^−/−^) were generated as previously described [5]. Mice were housed and maintained under procedures approved by the Institutional Animal Care and Use Committee at Duke University and the Durham VA Medical Centers. Mouse embryonic fibroblasts (MEF) were isolated from mice and immortalized by the standard 3T3 procedure as previously described [28]. Phoenix-eco cells used for packaging ecotropic retroviruses, as described below, are commercially available (Life Technologies, Grand Island, NY). All primary cells and cell lines were grown and maintained in a humidified atmosphere of 5% CO_2_ at 37°C in Dulbecco’s Modified Eagle Medium (DMEM, GIBCO, Life Technologies) supplemented with 2 mM L-glutamine, 4.5 g/L D-glucose, 110 mg/L sodium pyruvate, 10% (v/v) fetal bovine serum (FBS, HyClone, Logan, UT), 100 units/ml penicillin and 100 µg/ml streptomycin (GIBCO, Life Technologies). Where appropriate, 100 U/ml interferon (IFN)-γ (Calbiochem, EMD Biosciences, San Diego, CA, USA) was included in the growth medium.

### DNA Constructs, Mutagenesis, Transfection and Transduction

The mouse retroviral vector pRV-GFP was a gift from Dr. Carl Feng (sequence deposited with Addgene). Plasmids pGW1H/Irgm1 and pGW1H/Irgm1(ins 363,357E) were a gift from Dr. Jonathan Howard and have been described previously [17]. All restriction endonucleases were obtained from New England Biolabs (Ipswich, MA). To construct pRV-GFP/Irgm1, the full-length Irgm1 coding sequence was excised from another plasmid (pCWX200/Irgm1) as a BamHI/XhoI fragment and inserted into BglII and XhoI sites of pRV-GFP, under control of the viral LTR and upstream of the IRES and GFP cassettes. Mutagenesis of putative palmitoylation sites in Irgm1 retroviral and plasmid constructs was done using a site-directed mutagenesis kit (QuickChange II XL, Agilent Technologies, Santa Clara, CA) following the manufacturer’s protocol. Mutagenic oligonucleotides were obtained from Integrated DNA Technologies (Coralville, IA) - (Irgm1 (C8A): 5′catcacacagttccgccgaggctgctccac-3′, 5′-gtggagcagcctcggcggaactgtgtgatg-3′; Irgm1 (C257/258A): 5′-ctccaacatcagggccgctgaacccttaaagac-3′, 5′-gtctttaagggttcagcggccctgatgttggag-3′; Irgm1 (C371/373/374/375A): 5′-gagatttctcccagccgtagccgctgctttaagacgcttg-3′, 5′-caagcgtcttaaagcagcggctacggctgggagaaatctc-3′). The DNA sequences of all plasmid and mutant constructions were verified at the Duke University DNA Sequencing Facility (Durham, NC).

Transfection of primary MEFs and cell lines was done using XtremeGENE 9 reagent (Roche, Indianapolis, IN) following the manufacturer’s recommendations. For transduction of 3T3 cells, overnight cultures of Phoenix-eco cells seeded at a density of 3×10^6^ cells in 100 mm tissue culture dishes were transfected with either pRV-GFP/Irgm1 or one of the three putative palmitoylation site mutants. Culture supernatants containing packaged virus were collected on days 2 and 3 after transfection, pooled and used for infection of target cells. Irgm1^−/−^3T3 cells were grown to approximately 60% confluence in 25 cm^2^ tissue culture flasks or 6-well tissue culture plates. Virus culture supernatants were mixed 1∶2 with fresh medium. DEAE dextran (Sigma, St. Louis, MO) and polybrene (Sigma) were added to final concentrations of 2.5 µg/ml and 1 µg/ml respectively, and the virus-containing medium was added to the cells. After 6 h incubation at 37°C, the cultures were removed and centrifuged at 2000 rpm for 1 h in a Beckman GPR centrifuge equipped with microplate carriers and a swinging-bucket rotor. The flasks were then returned to the incubator. After overnight incubation, the medium was replaced and the transduced cells incubated for two days prior to use in experiments or splitting for expansion. GFP expression was monitored using an Olympus IMT-2 inverted fluorescence microscope to assess transduction efficiency.

### Antibodies

A rabbit polyclonal antibody (3266 [5]) and a mouse monoclonal antibody (1B2 [16]) to Irgm1 have been described previously. In the studies described here, 3266 was used for immunoprecipitations and immunoblotting; 1B2 for immunofluorescence. Rabbit monoclonal antibody EP892Y (Abcam, Cambridge, MA) to the Golgi matrix protein GM130, and rabbit polyclonal antibody FL-145 (Santa Cruz Biotechnology, Dallas TX) to mitochondrial outer membrane protein TOM20 were also used for immunofluorescence.

### Radiolabeling, Immunoprecipitation and Western Blotting

Wild-type or transduced Irgm1^−/−^3T3 cells grown in 6-well tissue culture plates were treated with 100U/ml IFN-γ for 24 h. Cells were pre-incubated in DMEM-10% dialyzed FBS (Sigma, St. Louis, MO) for 45 minutes prior to radiolabeling. To radiolabel the cells, an appropriate volume of stock ^3^H-palmitic acid [9,10-^3^H(N), 32.4 Ci/mmol, 5 mCi/ml, Perkin Elmer, Waltham, MA] in ethanol was removed and reduced by approximately 70% in a Speed-Vac concentrator (Savant Instruments, Farmingdale, NY) for 10–15 minutes. The concentrated stock solution was added to an appropriate volume of DMEM-1% dialyzed FBS to give a final concentration of 0.25 mCi/ml. Cells were incubated in the ^3^H-palmitate containing medium for 4 hours at 37°C. The radiolabeled cells were washed 3 times with ice-cold PBS and solubilized in 0.3 ml lysis buffer [1% (v/v) NP40 in PBS with proteinase inhibitors (Calbiochem, EMD)]. Lysates were centrifuged at 16,000×g for 5 minutes at 4°C to remove nuclei and insoluble debris. A 20 µl aliquot of each cleared lysate was removed to determine protein concentration using Precision Red reagent (Cytoskeleton, Inc., Denver, CO). Lysates were adjusted to the same protein concentration with lysis buffer and 16.25 µl removed for immunoblotting. The remaining lysate was used for immunoprecipitation. For each immunoprecipitation, 50 µl of protein G-coupled paramagnetic beads (Dynabeads, Invitrogen, Life Technologies, Carlsbad, CA) were incubated with the 3266 anti-Irgm1 antibody or pre-immune serum in 0.2 ml of PBS containing 0.02% Tween 20 (PBS-T) for 10 minutes at room temperature. Beads were kept in constant suspension during incubations. After separation on a magnetic stand (Promega, Madison WI), the antibody bound beads were washed once with PBS-T and the radiolabeled lysate added, followed by a 10 min incubation at room temperature. The beads were washed 4 times with PBS-T and suspended in 30 µl 1× LDS sample buffer with 0.1 M dithiothreatol (Invitrogen, Life Technologies). Proteins were removed from the beads by heating to 100°C for 5 minutes. Samples were separated on 8–16% Tris-acetate polyacrylamide gels (Invitrogen, Life Technologies), with equal amounts used for either immunoblot analysis or autoradiography. Lysate aliquots removed prior to immunoprecipitation were also included in the gel. Proteins were transferred to PVDF membranes (Millipore, Billerica, MA), and the membranes cut into sections for autoradiography or western blot analysis. The section containing samples for autoradiography was air dried for 2.5 hours at room temperature. Autoradiography film (Kodak BioMax, Carestream Health, Rochester, NY) was placed on the dried membrane with the emulsion side next to the samples, and then exposed for a minimum of 8 weeks in a light-tight film cassette with intensifying screens at −80°C. Membrane sections designated for western blot analysis were blocked in 5% (w/v) milk in TBS-Tween 20 (150 mM NaCl, 10 mM Tris-HCl pH 8.0, 0. 05% Tween-20) for 60 min, then incubated in the 3266 anti-Irgm1 antibody at 1∶1000 for 60 min, washed, and incubated in HRP conjugated secondary antibody (Clean-Blot, Thermo Scientific, Waltham, MA) at 1∶100 for 60 min. Washed blots were dipped in chemiluminescent substrate (SuperSignal West Pico, Thermo Scientific) and imaged on a Kodak Image Station 4000R using Carestream Molecular Imaging software.

### Immunofluorescence and Co-Localization

Primary WT and Irgm1^−/−^ MEF and were grown at a density of 2×10^4^ cells on poly- D-lysine-coated glass coverslips in 24 well tissue culture plates. When appropriate, the Irgm1^−/−^ MEF were transfected with the plasmids indicated in the text. All cells were treated with 100 U/ml IFN-µ for 24 hours prior to fixation. Cells were fixed in 4% paraformaldehyde (Sigma) in PBS for 15 minutes, permeabilized with 0.2% saponin (Sigma) in PBS for 10 minutes, and blocked for a minimum of 60 minutes in 0.2% saponin/PBS with 10% FBS. Cells were stained with primary antibodies for 60 minutes, washed 3 times with 0.2% saponin, and stained with appropriate fluorochrome-tagged secondary antibodies (Molecular Probes, Life Technologies, Eugene, OR) for 60 minutes. Monoclonal antibody 1B2 (Irgm1) was used as an undiluted hybridoma culture supernatant with saponin added to 0.2%. Primary antibodies EP892Y (GM130) and FL-145 (TOM20) were used at a dilution of 1∶250 in blocking buffer. Secondary antibodies were used at a dilution of 1∶750 in blocking buffer as recommended by the supplier. Cells were imaged on an Olympus IX70 inverted fluorescence microscope equipped with a Hamamatsu C8484-03G01 digital camera and ASI MS2000 XY Piezo Z stage. Images were collected as z-stacks with a plane thickness of 0.2 µm using Metamorph version 7.7.5.0. Z-stacks were deconvolved using Auto Quant X3 software. Co-localization measurements were performed using the Metamorph co-localization application. For colocalization analyses, in at least some experiments, the images were randomized by an independent party, and then assessed blindly. The co-localization analyses were performed on the indicated number of cells within each experiment; the average values from each of three separate experiments were then averaged to produce the displayed values.

### Treatment of Cells with 2-bromopalmitate

Wild-type 3T3 cells were grown to near confluence on polylysine-coated glass coverslips. After 24 h of treatment with 100U/ml IFN-γ the medium was replaced with normal medium containing 0.1 mM 2-bromopalmitate (Sigma) for 2 hours. Cells were washed with PBS, fixed and stained for immunofluorescence.

### S100 Fractionation of Membrane-Bound and Cytosolic Proteins

Irgm1^−/−^3T3 cells were transfected by electroporation with either pGW1H/Irgm1, pGW1H/Irgm1(ins 362,367E), pGW1H/Irgm1(C371/373/374/375A), or pGW1H/Irgm1(ins 362,367E, C371/373/374/375A). Each electroporation consisted of 10^6^ cells and 3 µg plasmid DNA, and cells were plated on a 35 mm tissue culture dishes. Electroporated cells were allowed to recover 7 hours at 37°C prior to the addition of 100U/m IFN-γ for 16 to 18 hours. Cell layers were washed twice with ice-cold PBS and scraped into a minimal volume of 200–250 µl homogenization buffer (HB, 8.5% sucrose 20 mM Tris pH 7.4). Cells were homogenized by passing through a 27ga needle fitted to a 1 ml syringe 10 times. Nuclei and large debris were pelleted by centrifugation at 735×g for 5 minutes at 4°C. Supernatants were removed and protein concentrations determined as described above. Volumes were adjusted to equal protein concentrations and equal volumes were loaded into ultracentrifuge micro tubes (Beckman, Palo Alto, CA) and centrifuged at 100,000×g for 60 minutes at 4°C in a TL-100 ultracentrifuge using a TLA 100.3 rotor (Beckman). Supernatants containing the cytosolic fraction were removed to a fresh tube, and pellets were resuspended in an equal volume of HB plus proteinase inhibitors. Complete resuspension of the pellets was accomplished by passage through a 27ga needle 10 times. LDS 4x sample buffer and 10x DTT were added to the supernatant and resuspended pellet fractions to a concentration of 1x. Samples were electrophoresed and subjected to western blot analysis as described above.

## Results

### Irgm1 is Palmitoylated

Membrane association of proteins is commonly mediated by lipid modifications that include myristoylation, prenylation, and palmitoylation. Myristoylation [29] and prenylation [30] take place at well-defined consensus sequences that are lacking in Irgm1. In contrast, palmitoylation occurs on cysteines in more variable sequence contexts that can, nevertheless, sometimes be predicted with computer algorithms developed using sequence data from a wide variety of palmitoylated proteins [31], [32]. Using one of these algorithms (CSS-Palm 2.0 [33]), we found that Irgm1 is potentially palmitoylated at any of seven different cysteines in the protein: Cys8, 257, 258, 371, 373, 374, and/or 375. Palmitoylation of Irgm1 in general has also been suggested in a recently published report identifying Irgm1 as an S-acylated protein in a proteomic screen, though the modification was not confirmed [34].

**Figure 1:**
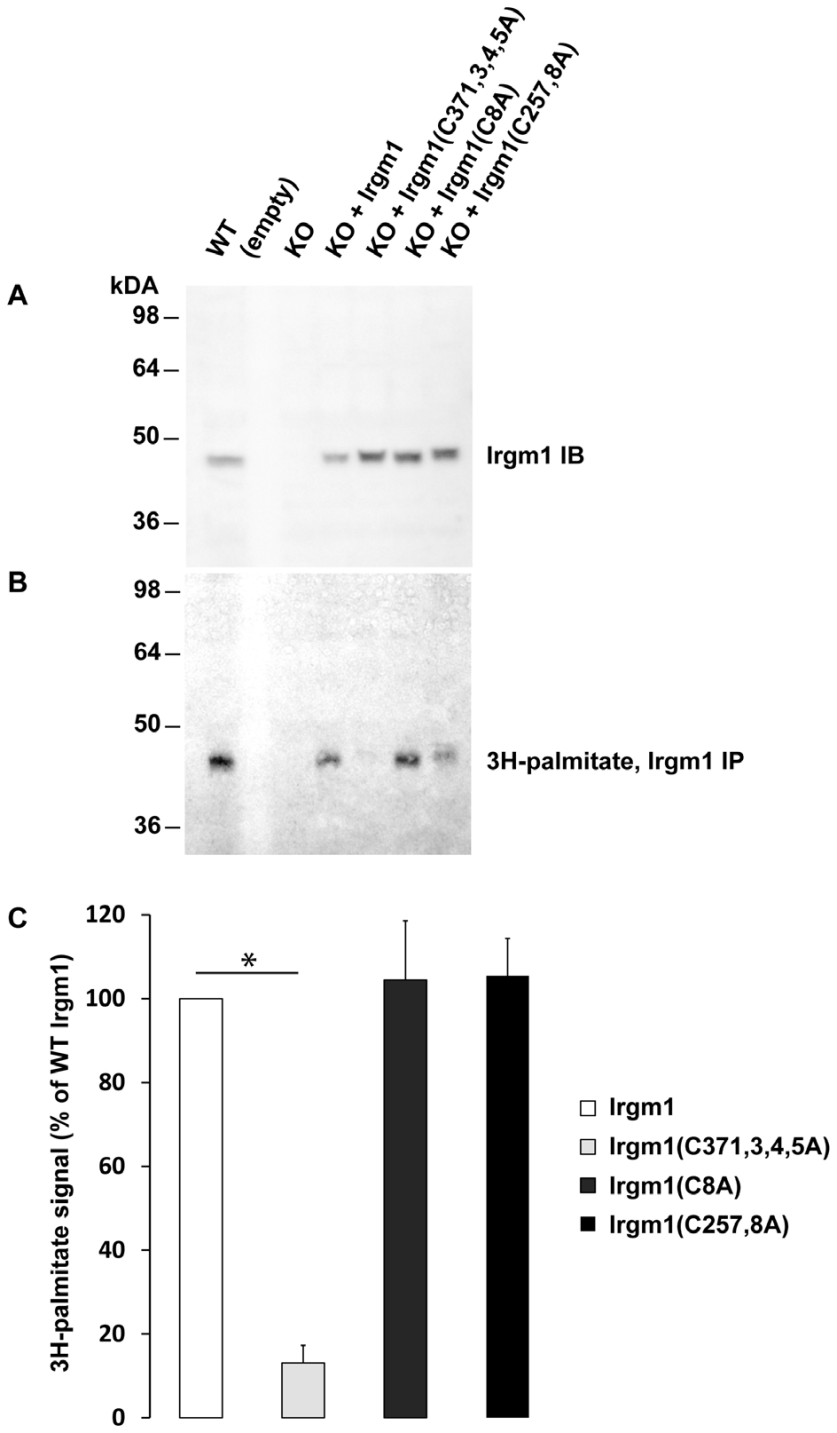
Palmitoylation of Irgm1. WT 3T3 MEF, Irgm1 KO 3T3 MEF, or Irgm1 KO 3T3 MEF stably transduced with the indicated Irgm1 mutants were exposed to 100U/mL IFN-γ for 24 h, and then incubated with ^3^H-palmitate for 4 h. Lysates were prepared and used for (**A**) 10% SDS-PAGE and western blotting with anti-Irgm1 antibodies, or (**B**) immunoprecipitation (IP) with anti-Irgm1 antibodies followed by 10% SDS-PAGE and autoradiography. Shown are representative results selected from 3 separate experiments. The positions of MW markers are shown at the left. Palmitoylation was quantified in each of the three immunoprecipitation studies, expressed as a value for each Irgm1 mutant relative to the value for WT Irgm1, and then average values across the three experiments displayed in (**C**), with error bars indicating standard error of the mean, and * indicating p<0.05 as determined using a one-sided z-distribution that was corrected for multiple comparisons.

To determine whether Irgm1 is palmitoylated in vivo, 3T3 fibroblasts were exposed to interferon (IFN)-γ to induce Irgm1 expression, pulse-labeled with [^3^H]-palmitate to radioactively label palmitoylated proteins, and then used for Irgm1 immunoprecipitation. A labeled Irgm1 band was clearly seen in WT but not Irgm1 KO cells (Fig. 1), indicating that the protein was palmitoylated. To address which cysteines were modified, cDNAs were generated that encoded mutant proteins with alanine substitutions at the three clusters of cysteines predicted to be palmitoylated: Irgm1 (C8A), Irgm1 (C257,258A), and Irgm1 (C371,373,374,375A). (These constructions and all others used in these studies were based on the full-length Irgm1 transcript, and not the recently described alternative transcript that contains a short amino-terminal truncation [35].) These Irgm1 mutants expressed in Irgm1 KO 3T3 fibroblasts were produced at approximately equal levels (Fig. 1A). In cells pulse-labeled with [^3^H]-palmitate, wild-type Irgm1, Irgm1(C8A), and Irgm1(C257,258A) all displayed roughly equal [^3^H]-palmitate labeling (Fig. 1B and 1C); in contrast, Irgm1(C371,373,374,375A) displayed little no detectable labeling (Fig. 1B and 1C), indicating that the predominant sites of Irgm1 palmitoylation are within the tight cluster of C371,373,374,375 near the C-terminus of the protein.

### Effect of Palmitoylation on Irgm1 Membrane Binding

**Figure 2:**
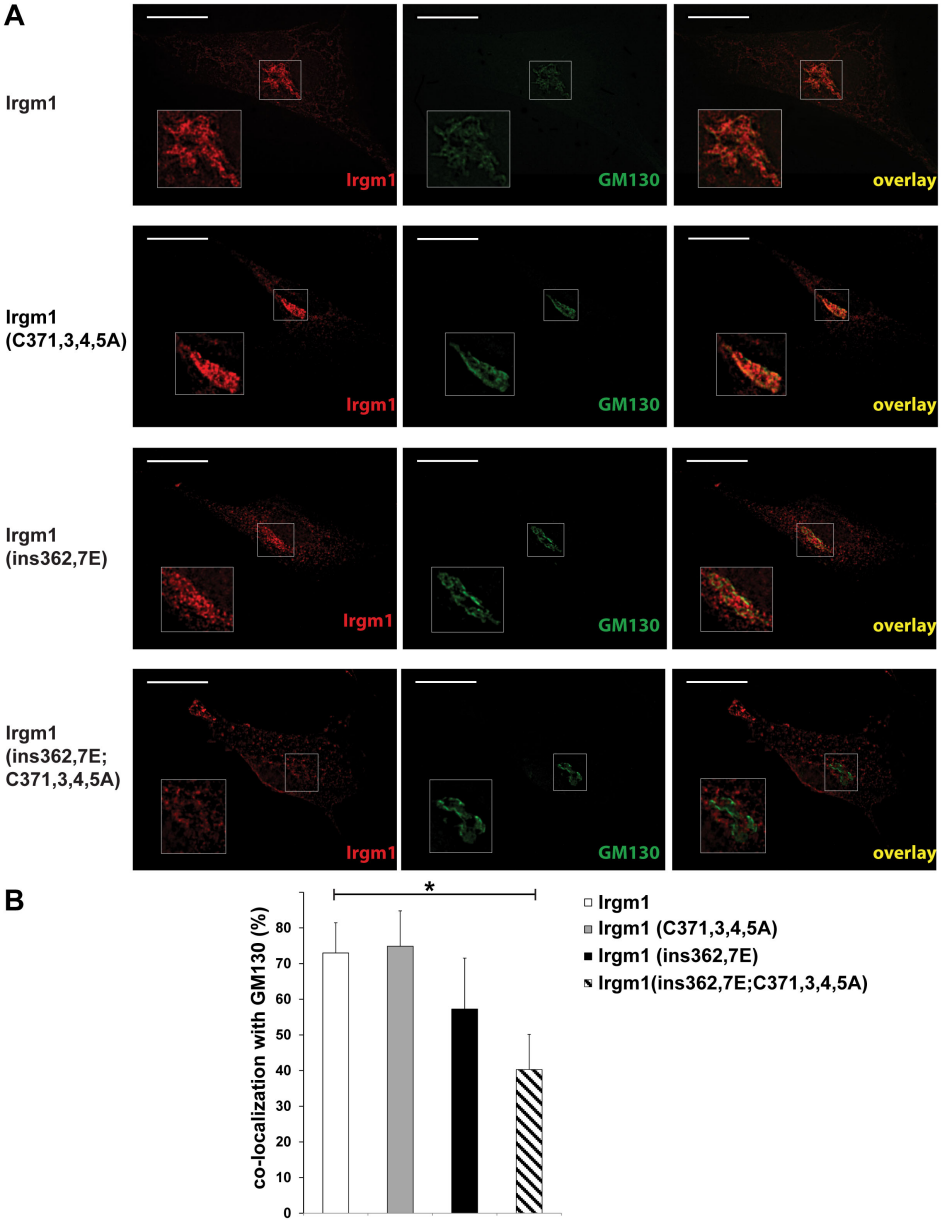
Effect of Irgm1 palmitoylation mutation on Golgi association. Irgm1 KO MEF were transfected with plasmids expressing wild-type or mutant Irgm1 proteins, as indicated. The cells were exposed to 100 U/ml IFN-γ for 24 h, stained with anti-Irgm1 and anti-GM130 antibodies, and used for immunofluorescence analysis. The experiment was performed 3 times, with at least 20 cells analyzed per group in each experiment. (**A**) Shown are images from representative cells. The scale bar represents 20 µm. (**B**) As detailed in the EXPERIMENTAL PROCEDURES, co-localization analysis was performed to quantify overlap between the Irgm1 and GM130 signals. In each experiment, the degree of co-localization was averaged for cells within an experimental group, and these values were then averaged across the three experiments, with error bars representing standard error the mean, and * representing p<0.05 as assessed by Student’s t-test.

**Figure 3:**
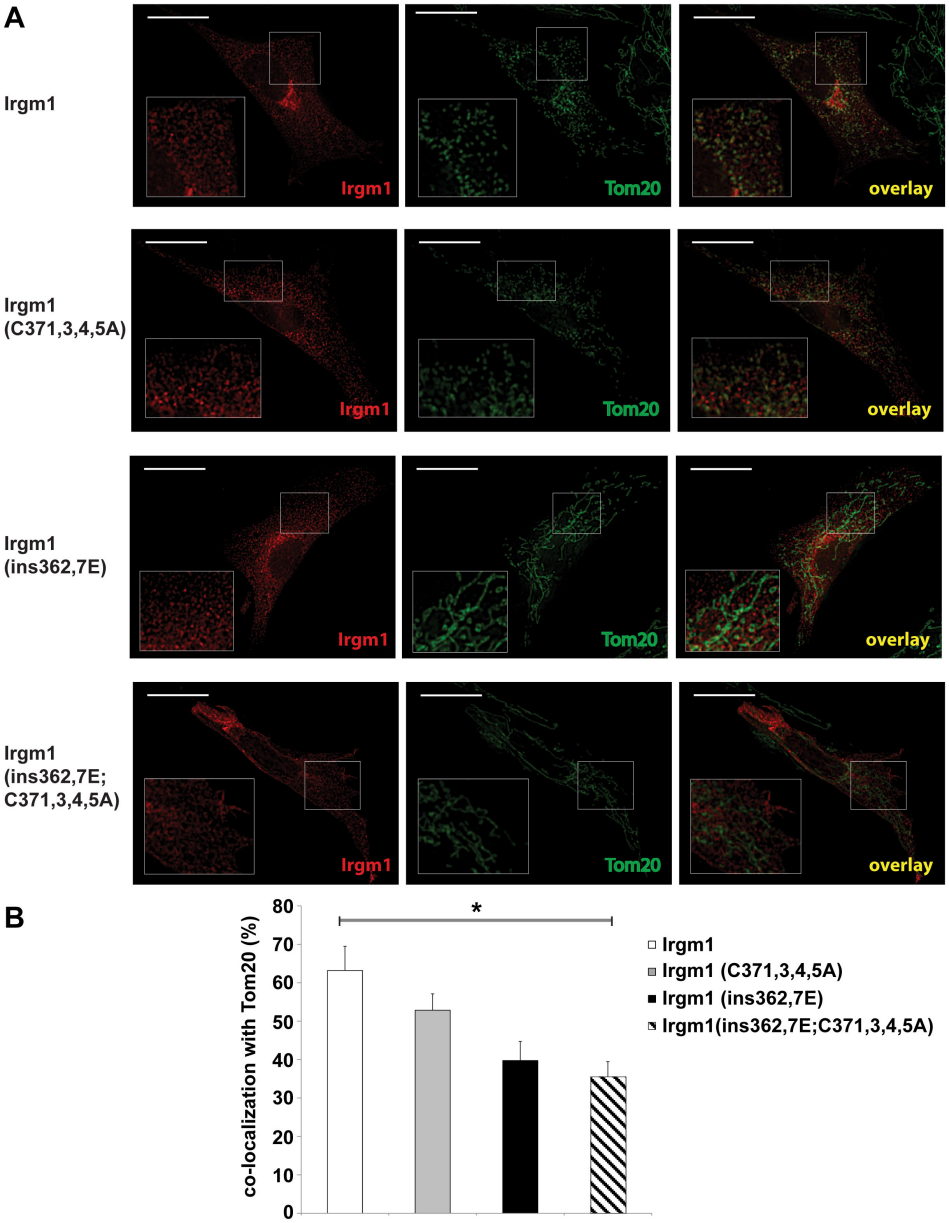
Effect of an Irgm1 palmitoylation mutation on mitochondrial association. Irgm1 KO MEF were transfected with plasmids expressing wild-type or mutant Irgm1 proteins, as indicated. The cells were exposed to 100 U/ml IFN-γ for 24 h, stained with anti-Irgm1 and anti-Tom20 antibodies, and used for immunofluorescence analysis. The experiment was performed 3 times, with at least 20 cells analyzed per group in each experiment. (**A**) Shown are images from representative cells. The scale bar represents 20 µm. (**B**) As detailed in the EXPERIMENTAL PROCEDURES, co-localization analysis was performed to quantify overlap between the Irgm1 and Tom20 signals. In each experiment, the degree of co-localization was averaged for cells within an experimental group, and these values were then averaged across the three experiments, with error bars representing standard error the mean, and * representing p<0.05 as assessed by Student’s t-test.

Palmitoylation mediates association of proteins with membranes, thereby impacting diverse aspects of their function (for reviews see [31], [32]). The palmitoyl group may function independently to mediate membrane binding, or in combination with other protein modifications such as myristoyl or prenyl modifications, or protein structural domains that interact with membranes such as amphipathic α-helices. We addressed whether the presence of the palmitoyl modification in Irgm1 played a role in its association with two of the predominant intracellular membrane compartments to which the protein has been found to localize: the Golgi apparatus (Fig. 2) and mitochondria (Fig. 3).

Wild-type Irgm1 and the palmitoyl mutant [Irgm1(C371,373,374,375A)] were expressed in Irgm1-deficient fibroblasts, and those cells were then used for immunofluorescence analysis. Extensive analysis of transfected cells suggested that Irgm1(C371,373,374,375A) localized well to the Golgi, with no substantial difference in the localization pattern compared to that of WT Irgm1 (Fig. 2A). These results contrasted with those for Irgm1 containing an insertional disruption of the predicted amphipathic α-helix, αK, which spans residues 356–369 in the C-terminal portion of Irgm1, and has previously been shown to be required for efficient Golgi association in cells [17], and for association with phosphatidylinositol lipids [PtdIns(3,4)P2 and PtdIns (3,4,5)P3] in *in vitro* binding assays [27]. That mutant - Irgm1(ins362,367E) - displayed an obvious reduction in association with the Golgi apparatus; however, there was substantial residual localization with the Golgi apparatus, or conceivably a shift to another compartment that interdigitates with the Golgi. A third mutant was analyzed that contained both the palmitoylation and αK mutations: Irgm1(ins362,367E;C371,373,374,375A). The images of this Irgm1 mutant displayed a nearly complete lack of apparent localization with the Golgi. Quantitative colocalization analysis was also performed with the same Irgm1 mutant proteins, with the results similarly indicating a loss of localization to the Golgi for the Irgm1(ins362,367E) mutant that was more pronounced with the Irgm1(ins362,367E;C371,373,374,375A) mutant (Fig. 2B). In general, the quantitative colocalization analysis indicated more residual colocalization than was apparent in visual inspection of the cell images, likely reflecting the diffuse nature of Irgm1 staining that contributes to background signal.

Regarding localization to mitochondria, WT Irgm1 typically displays a very punctate staining pattern that overlays or closely opposes discontinuous areas of those organelles ([27], [36], Fig. 3A). The Irgm1(C371,373,374,375A) palmitoylation mutant showed nearly as strong localization to the mitochondria as wild-type Irgm1 (Fig. 3A), although a small but reproducible loss of localization in the mutant was measured by colocalization analysis (Fig. 3B). The Irgm1(ins362,367E) mutant lacking a functional αK domain displayed a more obvious decrease in localization, by both subjective image analysis (Fig. 3A) and quantitative colocalization analysis (Fig. 3B). The decrease in localization was even more pronounced in the mutant lacking the palmitoylation and the αK domains Irgm1(ins362,367E;C371,373,374,375A), with little apparent mitochondrial localization remaining with subjective image analysis (Fig. 3A). Thus, as with the Golgi, the αK and palmitoylation domains cooperate to enable Irgm1 association with mitochondria.

**Figure 4:**
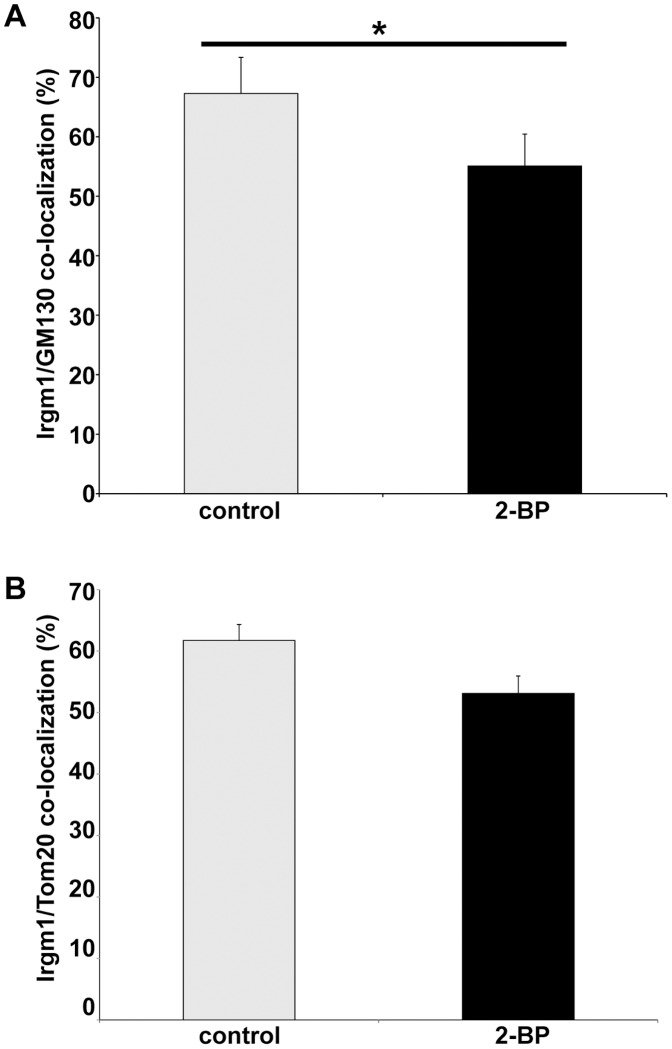
Effect of 2-bromopalmitate on Irgm1 association with the Golgi. WT MEF were exposed to 100/ml IFN-γ for 24 h, followed by exposure to the palmitoylation inhibitor, 2-bromopalmitate (0.1 mM), for 2 h. The cells were then co-stained with anti-Irgm1, and (**A**) anti-GM130 or (**B**) anti-Tom20 antibodies, and used for immunofluorescence analysis. Shown is the overlap between the Irgm1 and GM130 signals as determined by colocalization analysis, and expressed as an average from three (GM130) or four (Tom20) separate experiments, with between 25 and 59 cells analyzed per group in each experiment. The error bars represent standard error of the mean. The effect of 2-BP was significant in (A) (p = 0.009), but not (B) (p = 0.066).

An additional approach was undertaken to examine of palmitoylation of Irgm1. WT 3T3 cells were treated with 2-bromopalmitate (2BP), an inhibitor of palmitoylation [37], and then examined for the impact on Irgm1 localization to the Golgi (Fig. 4A) and mitochondria (Fig. 4B). 2-BP treatment produced a small decrease in Irgm1 localization to both the Golgi and mitochondria that was seen in all four repetitions of this experiment; however, the effect on localization was quite small, and in fact, considering inter-experimental variation was not statistically significant across the four studies in the case of mitochondrial co-localization. These studies edify the above mutant studies suggesting that palmitoylation of Irgm1 in itself has only a small, though reproducible, impact on localization of Irgm1 to the Golgi and mitochondria.

**Figure 5:**
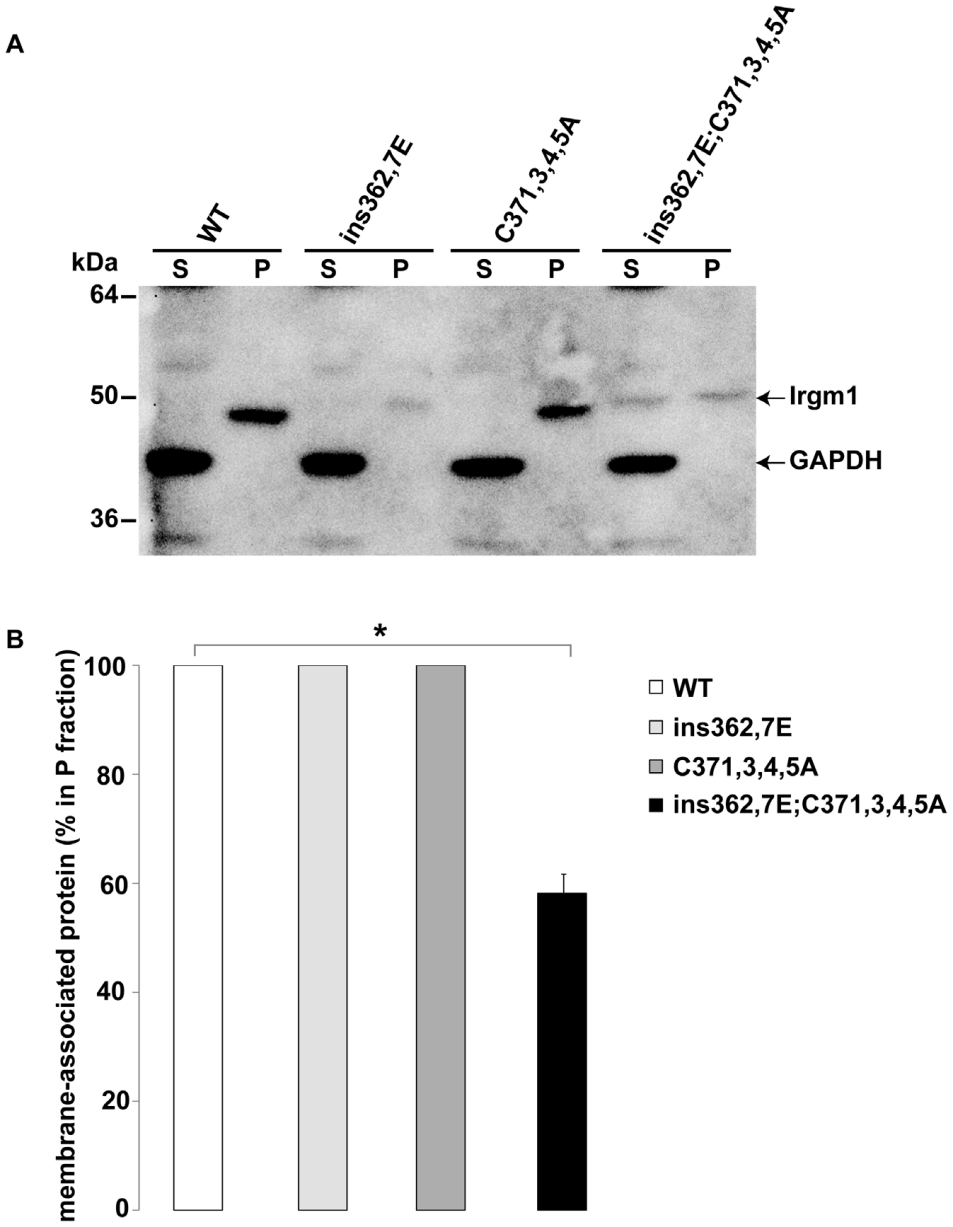
Effect of an Irgm1 palmitoylation mutation on membrane binding. Irgm1 KO MEF were transfected with plasmids expressing the indicated wild-type or mutant Irgm1 proteins. The cells were exposed to 100 U/ml IFN-γ for 16–18 h, and then used for preparation of detergent-free lysates that were separated into soluble (S) and membrane bound fractions (P) by centrifugation at 100,000×g. These fractions were separated on 10% SDS-PAGE gels that were used for western blotting with anti-Irgm1 and GAPDH antibodies. (**A**) Shown is a representative western blot, with the positions of MW markers shown at the left. (**B**) The percentage of the total protein that was detected in the P fraction was determined for three separate experiments. The average values are shown with error bars indicating standard error of the mean, and * indicating p<0.05 as determined using a one-sided z-distribution that was corrected for multiple comparisons.

In the above imaging experiments, while it was apparent that the combined palmitoylation and αK mutations led to loss of Irgm1 on the Golgi and mitochondria, Irgm1(ins362,367E;C371,373,374,375A) displayed residual association with the plasma membrane and unidentified punctate bodies in the cytoplasm (Figs. 2 & 3). To complement these observations and to confirm that the mutant protein maintained membrane binding, S100 fractionation was performed to separate cells lysed in the absence of detergent into membrane (P) and cytosolic (S) fractions (Fig. 5). WT Irgm1, Irgm1(C371,373,374,375A), and Irgm1(ins362,367E) were detected only in the membrane fraction and not in the cytosolic fraction, while in contrast, Irgm1(ins362,367E;C371,373,374,375A) was detected in both fractions with 58% on average in the membrane fraction. (Note that both of the insertional mutants lacking the αK motif consistently showed lower expression levels for reasons that were not determined. Additionally, the insertional mutants showed slightly slower mobility rates on SDS gels, again for reasons that were not determined but may relate to the slightly larger sizes of the proteins.) Taken together with the immunofluorescence data, these results suggest that when both the αK helix and palmitoylation motifs are lacking, about half of the cellular Irgm1 is released from membrane (particularly the Golgi and mitochondrial membranes), and the remaining half remains membrane-bound, though relocalized to the plasma membrane and other unidentified membrane compartments.

### Effect of Palmitoylation on Irgm1 Function

**Figure 6:**
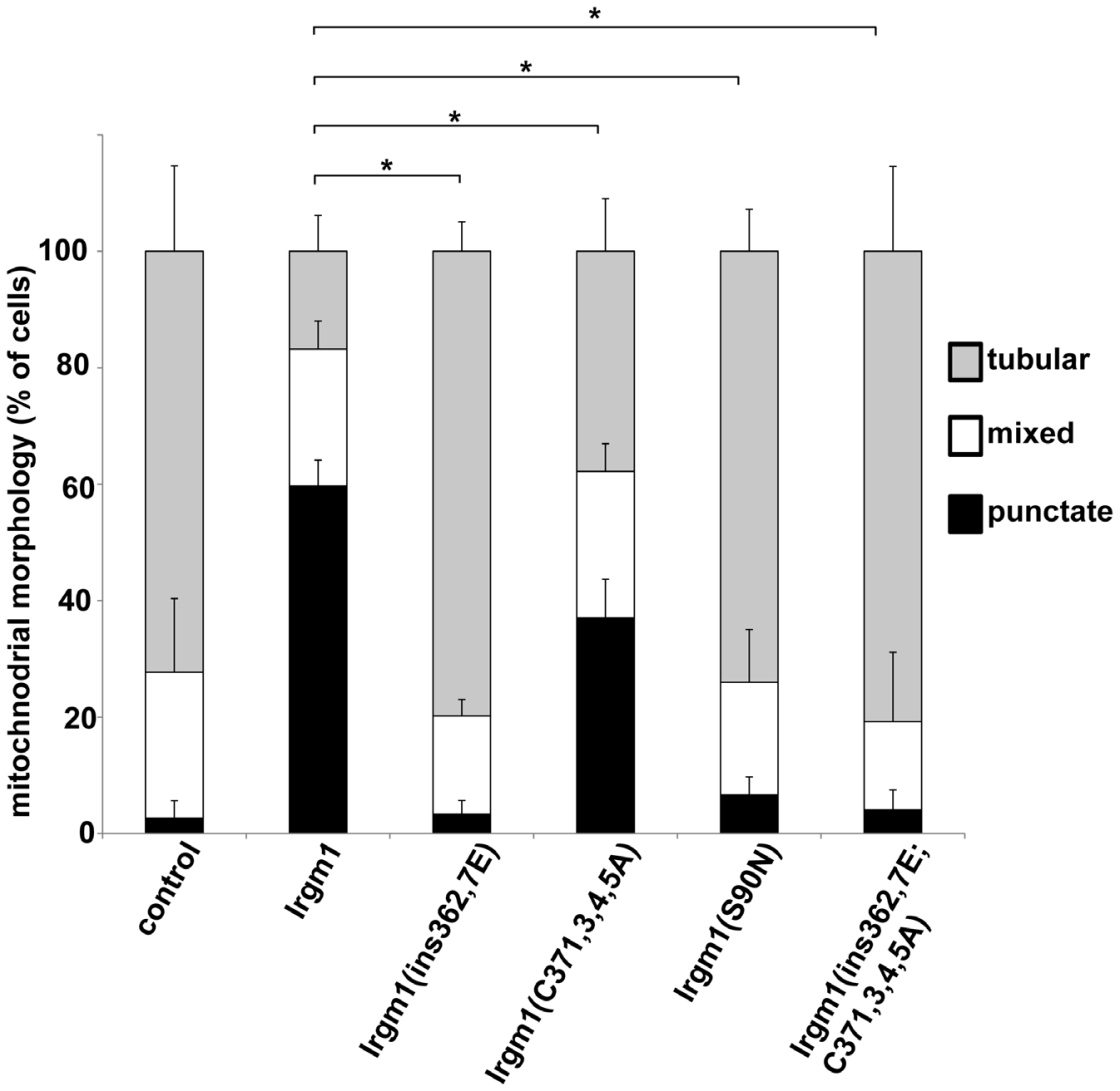
Effect of an Irgm1 palmitoylation mutation on the ability of the protein to promote formation of punctate mitochondria. Irgm1 KO MEF were transfected with plasmids expressing wild-type or mutant Irgm1 proteins, as indicated. The cells were exposed to 100 U/ml IFN-γ for 24 h, stained with anti-Irgm1 and anti-Tom20 antibodies, and used for immunofluorescence analysis. Images were collected from cells that expressed the Irgm1 proteins; the mitochondria in these images were scored in a blinded fashion as being punctate, tubular, or mixed phenotype. At least 20 cells were scored per experimental group, and the results displayed as percent of the total. Shown is the average of four separate studies, with error bars representing standard deviation, and * representing p<0.05 as assessed by Student’s t-test.

We next addressed whether palmitoylation was necessary for molecular functioning of Irgm1. Because the function of the protein in the Golgi has not been determined, we focused on its function in mitochondria. We have recently shown that absence of Irgm1 affects mitochondrial cycling between punctate and tubular forms, with the mitochondrial network in cells shifting toward tubular forms in absence of Irgm1 [38]. These results suggest that Irgm1 may drive mitochondrial fission, much has been shown for another dynamin-like GTPase, Drp1, which is known to act as a fission protein once it assembles on the mitochondrial membrane [16], [17]. To address potential activity for Irgm1, in the current studies we established that overexpression of Irgm1 in 3T3 fibroblasts promoted fission of mitochondria, pushing the mitochondrial equilibrium in the cell toward more punctate forms (Fig. 6). The palmitoylation mutant, Irgm1(C371,373,374,375A), displayed a modest but statistically significant decrease in its ability to shift the mitochondrial equilibrium toward punctate forms and away from tubular forms. The activity was more dramatically undermined in the αK mutant Irgm1(ins362,367E) and the combined αK/palmitoylation mutant Irgm1(ins362,367E;C371,373,374,375A). Additionally, an Irgm1 (S90N) mutant was tested that has a greatly reduced affinity for GTP and thus impaired GTPase functioning [17], [39]. The GTPase domain in IRG proteins has previously been shown to be necessary for their dimerization [23], and in the present studies, it was also required for the Irgm1-driven promotion of mitochondrial fission (Fig. 6). These results suggest that palmitoylation alone does have a small impact on the ability of Irgm1 to function in the mitochondria, while the αK motif has a more dominant effect (as does the GTPase activity of Irgm1). The data underscore that Irgm1 functioning in the mitochondria tracks closely with its ability to associate with mitochondrial membranes.

## Discussion

Although Irgm1 has been implicated in regulating several cellular functions that likely hinge on its ability to bind intracellular membranes, the biochemical mechanisms through which it does so are not entirely understood. In the work described above, we demonstrate that Irgm1 is palmitoylated, and we localize the sites of modification to a tight cluster of Cys (aa371, 373, 374, and 375) near the C-terminus of the protein that is immediately adjacent to αK (aa356–369), an amphipathic helix that was previously identified as a mediator of membrane binding [17], [27]. The proximity of the two regions is likely of functional relevance as palmitoylation, in general, can act in concert with other membrane binding motifs, including amphipathic α-helices to strengthen binding. (An example of another protein in which this occurs is the Regulator of G-protein signaling 4, RGS4 [40]). In these situations, the palmitoyl group is thought to add additional hydrophobic character to the hydrophobic face of the amphipathic helix. This may well be the case for Irgm1, though this cannot be addressed with certainty, as the structure of the protein has not been solved.

Irgm1 has been localized to several intracellular membrane compartments including the Golgi [16], [17], mitochondria [25], plasma membrane [17], lysosomes [20], phagosomes [6], [16], [17], and lipid droplets [23]. Previously published studies have shown that the αK mediates binding to the Golgi [17] and lysosomes [20]. The current work focused on the importance of palmitoylation in mediating binding to the Golgi - which seems to have the highest concentration of Irgm1 relative to the other membrane compartments – as well as to mitochondria given the potential importance of recent publications linking Irgm1 with the process of mitophagy [25]. Our data suggest that in both compartments, the αK provides an important anchor to the membrane, while the palmitoylation cooperates to strengthen this association, so that in absence of both motifs – but not in absence of them individually - there was little apparent residual localization to the Golgi and mitochondria. The palmitoylation domain, acting on its own, seems to have a small or no impact on the localization to these compartments. Further, the two membrane binding motifs impacted the function of Irgm1 in the mitochondria, with the relative impact of the two motifs on driving the Irgm1-mediated shift toward punctate mitochondrial forms paralleling their ability to drive membrane association. There are certainly precedents for palmitoylation mediating the binding of other proteins to the Golgi or mitochondria (e.g. [34], [41]). It is also notable that when the αK/palmitoylation signal of Irgm1 is lacking, a substantial proportion of the protein still resided on intracellular membranes but in other membrane compartments, the plasma membrane in particular. While this underscores the importance of the αK/palmitoylation signal in maintaining Golgi and mitochondrial binding, it suggests additional membrane binding signals are still present, and that these multiple signals may partition cellular Irgm1 to distinct membrane compartments to mediate different facets of Irgm1 function. It is also possible that some of the residual membrane binding may be indirect through association with other proteins, which in turn, directly bind to the membrane.

Among the IRGM subfamily of IRG proteins, palmitoylation may be specific to Irgm1, as the cluster of Cys that are palmitoylated in Irgm1 are absent in the mouse proteins Irgm2, Irgm3, and the human protein IRGM. It cannot be ruled out that non-conserved Cys are palmitoylated in these other IRGM proteins. However, compared to Irgm2 and Irgm3, Irgm1 has a unique localization profile within the cell [16], [17], so it is tempting to speculate that palmitoylation could play a role in establishing the distinct distribution of Irgm1 in mouse cells. Palmitoylation is also known to be a reversible modification for some proteins [42]: For instance, β-adrenergic receptor activation accelerates depalmitoylation of receptor-associated Gαs, shifting its localization to the cytoplasm [43]. This raises the additional possibility that the impact of palmitoylation on Irgm1 function could be dynamic and regulated, possibly directing the function of Irgm1 during infection or in other physiological contexts. It is also conceivable that palmitoylation of Irgm1 could be altered during infection, as the palmitoylation machinery of the cell is a common target for multiple pathogens [44]. These will be important areas for future research.

## Conclusions

Our studies establish that Irgm1 is palmitoylated. This modification, in itself, has a small impact on localization of the protein to the Golgi and mitochondria, and a small impact on promotion of mitochondrial fission by Irgm1. However, palmitoylated in combination with the αK amphipathic helix provides a major anchor for the protein on membranes of the Golgi apparatus and mitochondria, and allows Irgm1 to function in the mitochondria.
